# Upregulation of long non-coding RNA urothelial carcinoma associated 1 by CCAAT/enhancer binding protein α contributes to bladder cancer cell growth and reduced apoptosis

**DOI:** 10.3892/or.2014.3092

**Published:** 2014-03-19

**Authors:** MEI XUE, XU LI, WENJING WU, SHUWAN ZHANG, SHOUZHEN WU, ZHENGKUN LI, WEI CHEN

**Affiliations:** 1 Center for Translational Medicine, The First Affiliated Hospital, School of Medicine, Xi’an Jiaotong University, Xi’an 710061, P.R. China; 2 Clinical Laboratory, The First Affiliated Hospital, School of Medicine, Xi’an Jiaotong University, Xi’an 710061, P.R. China

**Keywords:** long non-coding RNA, urothelial carcinoma associated 1, bladder cancer, transcription factor, CCAAT/enhancer binding protein α

## Abstract

Long non-coding RNA urothelial carcinoma associated 1 (lncRNA-UCA1) is upregulated in bladder cancer and plays a pivotal role in bladder cancer progression and metastasis. Recent studies and our research found that lncRNA-UCA1 may be an important biomarker and therapeutic target for bladder cancer. However, the molecular mechanism involved in the upregulation of lncRNA-UCA1 in bladder cancer is largely unknown. In the present study, we showed that lncRNA-UCA1 expression in bladder cancer cells was upregulated by transcription factor CCAAT/enhancer binding protein α (C/EBPα), which was the only candidate transcription factor simultaneously predicted by a total of five bioinformatical software programs. Electrophoretic mobility shift assay and chromatin immunoprecipitation assay indicated that C/EBPα bound to the lncRNA-UCA1 core promoter region *in vitro* and *in vivo*. The luciferase assays further showed that there was a point mutation (A231G) in the C/EBPα binding site of the lncRNA-UCA1 core promoter in various bladder cancer cell lines, which in turn significantly increased the transcriptional activity of lncRNA-UCA1. We also demonstrated that C/EBPα siRNA treatment contributed to the downregulation of lncRNA-UCA1 expression, whereas overexpression of C/EBPα enhanced lncRNA-UCA1 expression. Furthermore, lncRNA-UCA1 transcriptional repression by C/EBPα siRNA sharply reduced cell viability and induced cell apoptosis *in vitro*. Collectively, our results provide a novel therapeutic strategy for bladder cancer by effectively interrupting the binding of the lncRNA-UCA1 promoter and certain transcription factors, so as to reverse the upregulation of lncRNA-UCA1 and prevent bladder cancer progression.

## Introduction

Bladder cancer is the most common malignancy in the male urinary system. In the United States, 72,570 new cases and 15,210 deaths from bladder cancer were estimated to occur in 2013 (1). Despite advances in surgical and chemical therapies, the high incidence of metastasis and recurrence accounts for the main cause of bladder cancer mortality (2). Therefore, efforts in exploring molecular markers and therapeutic targets against metastasis and relapse of bladder carcinoma are of particular significance (3). Long non-coding RNAs (lncRNAs, >200 nucleotides), a huge body of emerging transcripts incapable of coding proteins yet exhibiting multiple regulatory functions, have gained increasing attention in the field of molecular biology and have been found to be involved in diverse essential bioactivities including cell proliferation and apoptosis (4–6). A large number of complex human diseases, particularly cancers, are accompanied by abnormal lncRNA expression and consequent dysfunction (7). Previous research has shown that certain lncRNAs can be applied as novel biomarkers and targets for cancer detection and treatment (8).

Long non-coding RNA urothelial carcinoma associated 1 (lncRNA-UCA1) is a highly specific lncRNA exclusively expressed in bladder cancer, with significantly higher expression in bladder cancer tissues when compared with that in adjacent normal tissues (9). Detection of lncRNA-UCA1 in urine sediment has proven to be highly sensitive and specific for diagnosing bladder carcinoma. Ectopic lncRNA-UCA1 expression promotes the proliferation, motility, invasion and drug resistance of bladder cancer cells (10–12). Taken together, lncRNA-UCA1 is a potential biomarker for bladder cancer diagnosis and prognosis (13). However, the mechanism underlying the upregulation of lncRNA-UCA1 expression in bladder cancer remains to be elucidated. Although the biological origins of lncRNAs are extremely complicated, their transcriptional regulation and post-transcriptional processing are similar to those of protein-coding genes. A number of studies concerning the transcriptional regulation of lncRNAs indicate that transcription factors regulate lncRNAs mainly by binding with their promoters (14,15). Both lncRNAs and protein-coding genes are regulated by classic transcriptional regulatory proteins.

In the present study, we aimed to elucidate the mechanism underlying the upregulation of lncRNA-UCA1 in bladder cancer cells. We demonstrated that CCAAT/enhancer binding protein α (C/EBPα) binds to the core promoter region of lncRNA-UCA1 *in vitro* and *in vivo*. When the expression of lncRNA-UCA1 is upregulated by C/EBPα, it contributes to increased cell viability and reduced cell apoptosis *in vitro*. Our results, therefore, demonstrate a regulatory mechanism for lncRNA-UCA1 upregulation in bladder cancer cells. Knowledge of the mechanism involved in the upregulation of lncRNA-UCA1 will be of huge benefit for both basic research and therapeutic application in human bladder cancer.

## Materials and methods

### Cell culture and siRNA transfection

The human bladder cancer cell lines 5637 and T24 were obtained from the American Type Culture Collection (ATCC; Manassas, VA, USA). The human bladder cancer cell lines BLZ-211 and BLS-211 are described elsewhere (16). The cells were grown in RPMI-1640 (Gibco, Gaithersburg, MD, USA) with 10% bovine calf serum. The cultures were maintained at 37°C under a humidified 5% CO_2_ atmosphere. C/EBPα siRNA was transiently transfected into bladder cancer cell lines using X-tremeGENE siRNA transfection reagent (Roche Diagnostics, Indianapolis, IN, USA). Following 48 h of C/EBPα siRNA transfection, the cells were harvested for further studies. C/EBPα siRNAs (Shanghai Genepharma Co., Ltd., Shanghai, China) are listed in Table I.

### Transcription factor predictions

Transcription factor predictions were carried out using AliBaba (http://www.gene-regulation.com/pub/programs.html#alibaba2), Matrix Catch (http://www.gene-regulation.com/pub/programs.html#mcatch), TESS (http://www.cbil.upenn.edu/tess), TFSEARCH (http://www.cbrc.jp/research/db/TFSEARCH.html) and Patch (http://www.gene-regulation.com/pub/programs.html#patch) software programs with the default parameter values. The motifs of transcription factor C/EBPα that bind with the lncRNA-UCA1 promoter were determined according to JASPAR (http://jaspar.cgb.ki.se) database matrix information.

### Electrophoretic mobility shift assay (EMSA)

The nuclear proteins of bladder cancer cells were prepared using NE-PER nuclear and cytoplasmic extraction reagents (Pierce, Rockford, IL, USA). EMSA was carried out using a LightShift chemiluminescent EMSA kit (Pierce). The probes are listed in Table I.

### Chromatin immunoprecipitation assay (ChIP)

ChIP was performed using an EZ-ChIP chromatin immunoprecipitation kit (Millipore, Bedford, MA, USA). Chromatin was immunoprecipitated using anti-C/EBPα antibodies (Abcam, Cambridge, MA, USA). Human IgG was used as the negative control. The precipitated DNA was monitored by PCR using specific primers for the lncRNA-UCA1 promoter. The primers are listed in Table I.

### Quantitative real-time PCR

Total RNA was extracted from bladder cancer cell lines using TRIzol reagent (Invitrogen, Carlsbad, CA, USA). First-strand cDNA was synthesized using the PrimeScript RT reagent kit (Perfect Real-Time; Takara, Dalian, China). Quantitative real-time PCR was carried out using a SYBR Premix Ex Taq™ II (Takara) on a CFX96 real-time PCR system (Bio-Rad Laboratories, Hercules, CA, USA), and the results were normalized to β-actin as an internal control. The primers are listed in Table I.

### Plasmid constructs and transient transfection

The lncRNA-UCA1 promoter reporter constructs were obtained by PCR. The PCR products were digested and ligated into pGL3 basic vector. The C/EBPα binding site mutation was generated using a QuickChange Multi Site-Directed Mutagenesis kit (Stratagene, La Jolla, CA, USA). The lncRNA-UCA1 promoter reporter plasmid was used as the template. The mutant primers are listed in Table I. The human pGV219-C/EBPα expression vectors and empty vectors (GV219) were obtained from Shanghai Genechem Co., Ltd. (Shanghai, China). Bladder cancer cells were transiently transfected with a pGV219-C/EBPα or empty vector (GV219) as a control using the X-tremeGENE HP DNA transfection reagent (Roche). After 48 h of transfection, the cells were harvested for further studies.

### Luciferase reporter assay

Transient transfection of the lncRNA-UCA1 promoter reporter plasmid and the internal control *Renilla* luciferase plasmid was carried out with the X-tremeGENE HP DNA transfection reagent. After 48 h of transfection, luciferase activity was measured using a dual-luciferase reporter gene assay system (Promega, Madison, WI, USA).

### MTT assay

The cells were transfected with C/EBPα siRNA. Every day until day 6, MTT (Amresco, Solon, OH, USA) was added to each well, and incubation was carried out at 37°C for 4 h. The medium was removed and DMSO was added into each well. Absorbance was measured at 490 nm.

### Cell apoptosis assay

After 48 h of treatment with C/EBPα siRNA, the cells were stained with Annexin V-FITC and propidium iodide (Beyotime Institute of Biotechnology, Haimen, China) and examined using a flow cytometer (FACS; BD Biosciences, Sparks, MD, USA).

### Statistical analysis

All the experiments were performed at least in triplicate. Data are presented as means ± SEM, and were analyzed using the SPSS 19.0 and Graphpad Prism 5. Statistical analyses were carrried out using a two-tailed unpaired Student’s t-test. Differences with P<0.05 were considered statistically significant.

## Results

### Bioinformatic analysis of the lncRNA-UCA1 core promoter region

**Figure 1:**
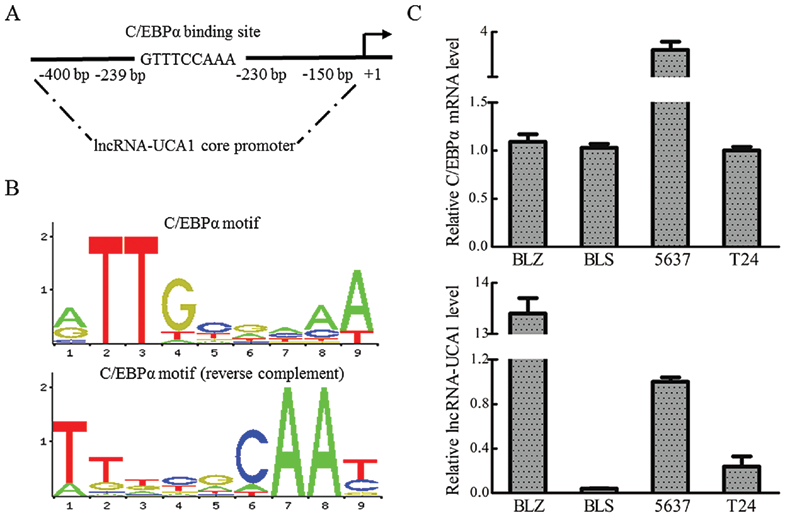
Bioinformatic analysis of the lncRNA-UCA1 core promoter region. (A) Schematic representation of the putative C/EBPα binding site in the lncRNA-UCA1 core promoter. (B) Sequence logo of C/EBPα was obtained from the JASPAR database. (C) C/EBPα and lncRNA-UCA1 expression levels were analyzed in the various cell lines by real-time PCR. β-actin was used as the internal control.

In our previous study, we confirmed that the lncRNA-UCA1 promoter was located at the 5′ end of the lncRNA-UCA1 gene, from −1800 bp to +200 bp, with the core promoter ranging from −400 bp to −150 bp (17). Several potential transcription factor binding sites were predicted in the lncRNA-UCA1 core promoter region. C/EBPα was the only candidate transcription factor predicted by five bioinformatical software programs simultaneously (Table II). It was further speculated that there were more than one putative C/EBPα binding site in the lncRNA-UCA1 core promoter region (Table III). Based on the JASPAR database, we determined that a unique motif of C/EBPα (from −239 bp to −230 bp, GTTTCCAAA) was potentially eligible to interact and bind with the lncRNA-UCA1 core promoter (Fig. 1A and B). In order to screen out a perfect cell model by which to validate our prediction results, we detected the constitutive expression of C/EBPα and lncRNA-UCA1 in three bladder cancer cell lines. As shown in Fig. 1C, C/EBPα was expressed in all of the three bladder cell lines. lncRNA-UCA1 expression was high in the cell line BLZ-211, yet extremely low, if not absent, in its counterpart cell line BLS-211, although the two cell lines were derived from the same patient (16). Therefore, BLS-211 was employed as the control cell line in the subsequent experiments.

### C/EBPα binds to the lncRNA-UCA1 core promoter

**Figure 2:**
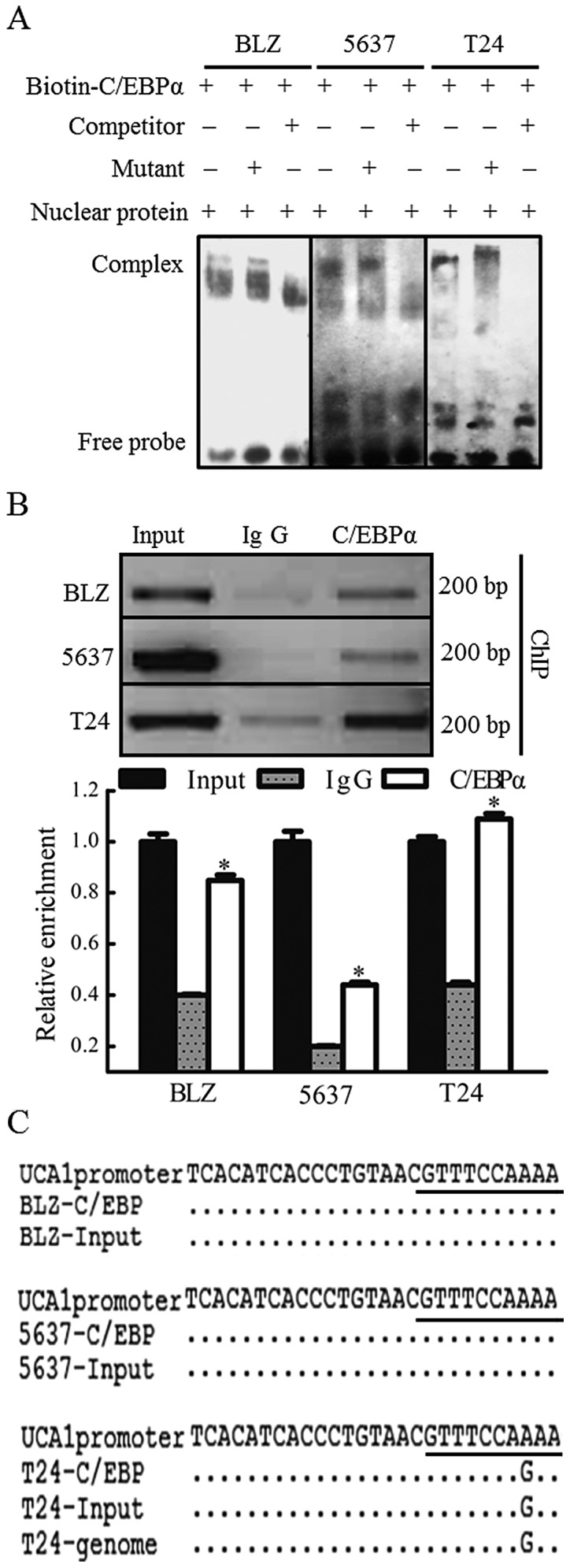
C/EBPα binds with the lncRNA-UCA1 core promoter. (A) EMSA indicates the interaction of C/EBPα with the lncRNA-UCA1 core promoter *in vitro*. (B) ChIP indicates the interaction of C/EBPα with the lncRNA-UCA1 core promoter *in vivo* (^*^P<0.05; n=3). (C) PCR products from the ChIP were sequenced. The sequences were aligned using BioEdit.

To confirm whether C/EBPα binds to the lncRNA-UCA1 core promoter region, we performed an electrophoretic mobility shift assay (EMSA) using biotin-labeled DNA probes with C/EBPα binding site sequences. The complexes of nucleoprotein and biotin-labeled DNA probes were detected in all the three cell lines, BLZ-211, 5637 and T24. However, no complexes in the control group with cold probes (unlabeled probes) competing with the biotin-labeled DNA probes were detected in the three cell lines. Furthermore, the mutant probes (C/EBPα binding sites mutant) were unable to competitively inhibit the formation of the complex bands and thus complexes were also detected in this group (Fig. 2A). Together, these results suggest that C/EBPα specifically interacts and binds with the lncRNA-UCA1 core promoter *in vitro*.

**Figure 3:**
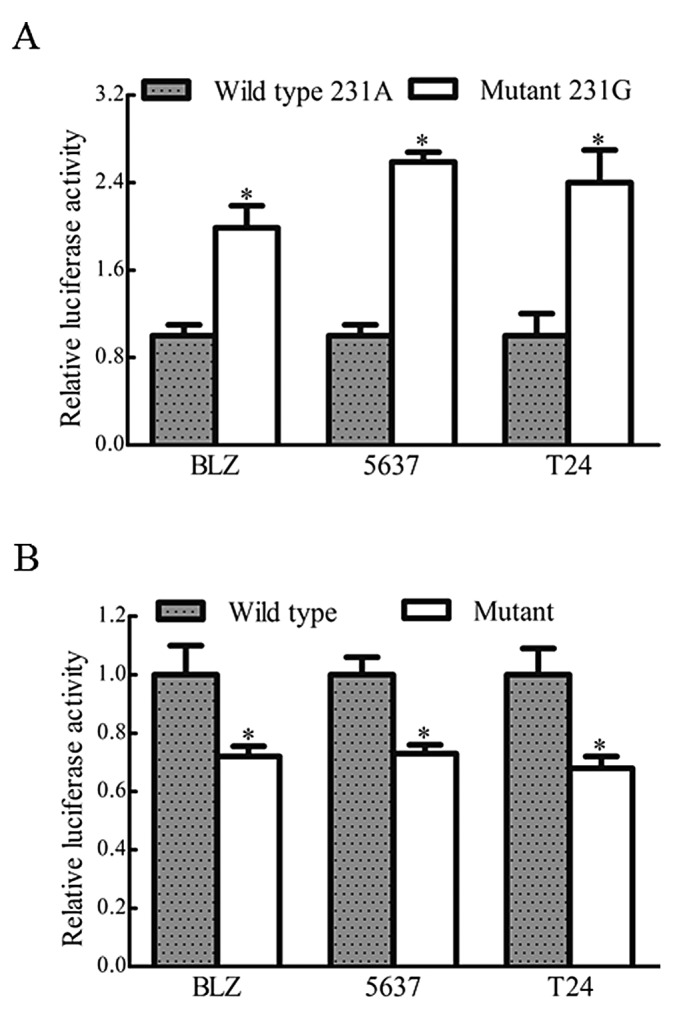
C/EBPα binding site contributes to lncRNA-UCA1 transcriptional activation. (A) Bladder cancer cells were transfected with C/EBPα binding site mutant (231G) lncRNA-UCA1 promoter constructs. lncRNA-UCA1 promoter activity was detected by luciferase assay (^*^P<0.05; n=3). (B) Bladder cancer cells were transfected with C/EBPα binding site deleted lncRNA-UCA1 promoter constructs. lncRNA-UCA1 promoter activity was detected by luciferase assay (^*^P<0.05; n=3).

We then performed ChIP experiments to further confirm the binding of C/EBPα to the lncRNA-UCA1 core promoter *in vivo*. As shown in Fig. 2B, C/EBPα was able to bind to the C/EBPα binding site (-239 bp to −230 bp) of the lncRNA-UCA1 core promoter in the BLZ-211, 5637 and T24 cells. Importantly, a base transversion (A231G) of the C/EBPα binding site sequence was found in the T24 cells (Fig. 2C). We then aimed to ascertain whether this point mutation affects the binding activity between C/EBPα and the lncRNA-UCA1 promoter. Thus, we were motivated to establish lncRNA-UCA1 promoter constructs with mutant (231G) and wild-type (231A) C/EBPα binding sites. The luciferase assay demonstrated that the mutant C/EBPα binding site (231G) had an increase in luciferase activity, when compared with that of the wild-type (231A) in all cell lines (~2-fold; Fig. 3A). Collectively, these data indicate that C/EBPα binds with the lncRNA-UCA1 core promoter *in vitro* and *in vivo*.

### C/EBPα regulates lncRNA-UCA1 expression

**Figure 4:**
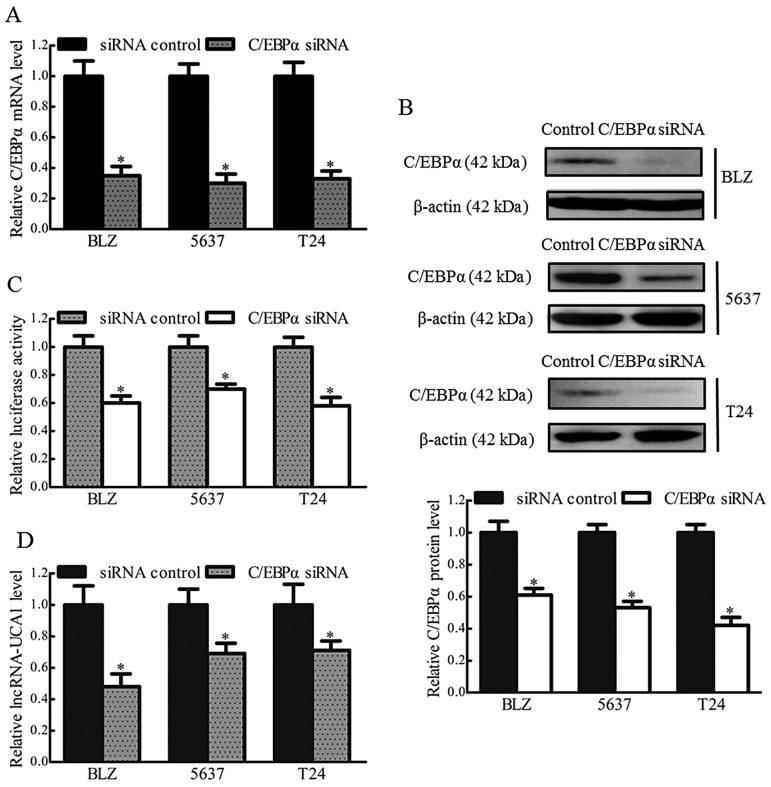
C/EBPα binds to the core promoter regions of lncRNA-UCA1 and regulates its expression. (A and B) Knockdown efficiency of C/EBPα siRNA was determined by real-time PCR and western blotting. β-actin was used as the internal control (^*^P<0.05; n=3). (C and D) The effects of C/EBPα silencing on the lncRNA-UCA1 promoter activity and expression were detected by luciferase assay and real-time PCR. β-actin was used as the internal control (^*^P<0.05; n=3).

**Figure 5:**
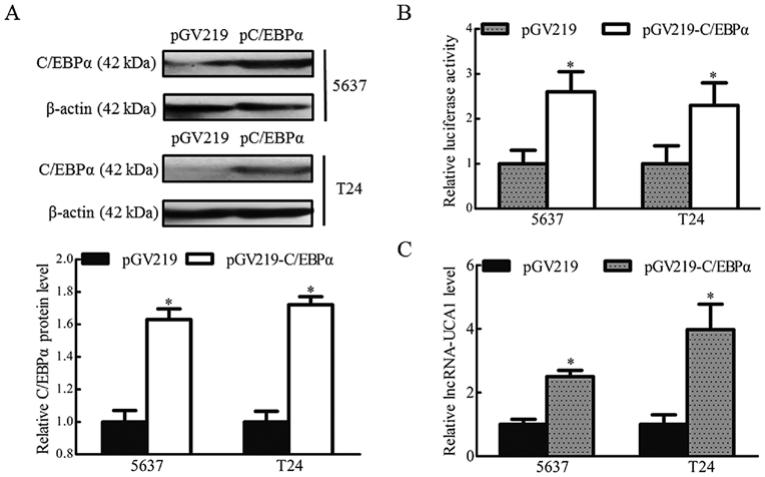
C/EBPα upregulates lncRNA-UCA1 expression. (A) Overexpression efficiency of the C/EBPα expression vector was detected by western blotting. β-actin was used as the internal control (^*^P<0.05; n=3). (B and C) The effects of C/EBPα overexpression on the lncRNA-UCA1 promoter activity and expression were detected by luciferase assay and real-time PCR. β-actin was used as the internal control (^*^P<0.05; n=3).

To verify whether C/EBPα regulates lncRNA-UCA1 transcription in bladder cancer cells, a C/EBPα binding site deleted lncRNA-UCA1 promoter construct (mutant) was cloned. The cells transfected with the mutant promoter construct displayed a ~30% reduction in luciferase reporter activities (Fig. 3B). Therefore, the C/EBPα binding site contributes to lncRNA-UCA1 transcriptional activation. BLZ-211, 5637 and T24 cells were transfected with C/EBPα siRNA. The C/EBPα mRNA and protein levels were reduced by ~70 and ~50%, respectively (Fig. 4A and B). Silencing of C/EBPα also reduced lncRNA-UCA1 promoter activities (30–50% reduction) and expression levels (30–50% reduction) in the BLZ-211, 5637 and T24 cells (Fig. 4C and D), while overexpression of C/EBPα in the 5637 and T24 cells led to an increase in C/EBPα protein levels (~1.65-fold; Fig. 5A), followed by increases in lncRNA-UCA1 promoter activity (~2.5-fold; Fig. 5B) and expression levels (~3-fold; Fig. 5C), respectively. Taken together, these results indicate that C/EBPα strengthens lncRNA-UCA1 transcription.

### C/EBPα regulates lncRNA-UCA1 expression to increase cell viability and reduce cell apoptosis

**Figure 6:**
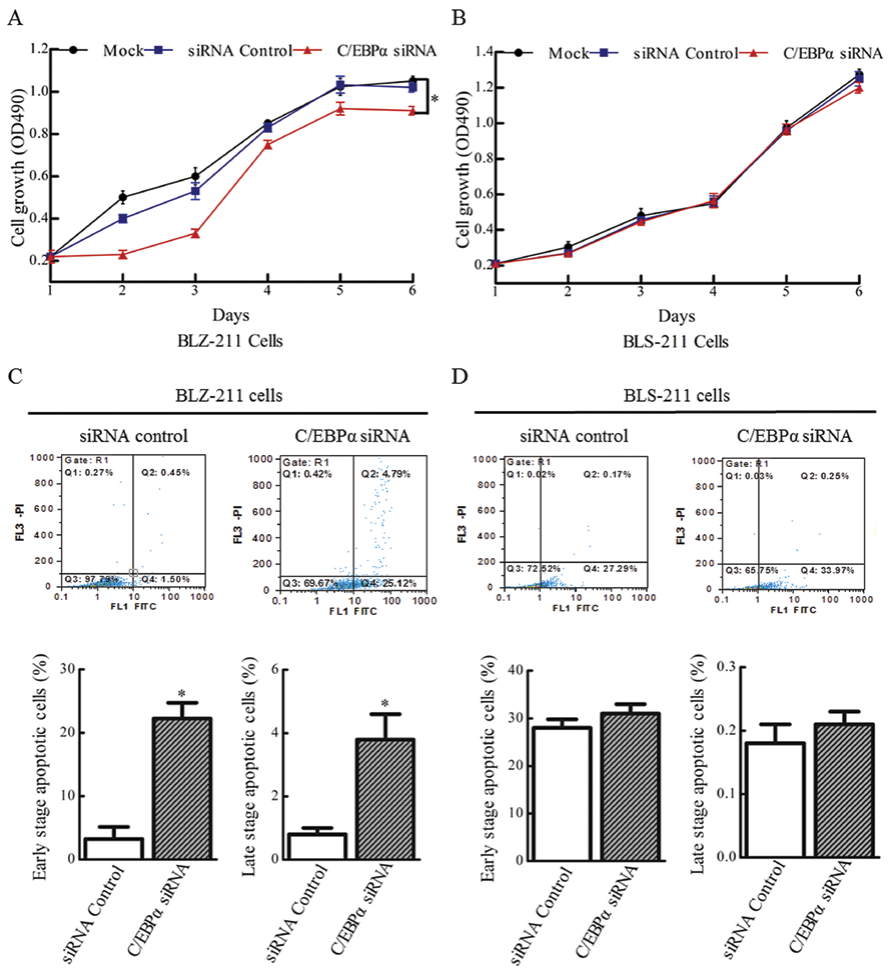
C/EBPα regulates lncRNA-UCA1 expression to increase cell viability and reduce cell apoptosis. (A and B) Bladder cancer cells were transfected with C/EBPα siRNA, and the cell viability was detected by MTT assay (^*^P<0.05; n=3). (C and D) Bladder cancer cells were transfected with C/EBPα siRNA, and apoptosis was detected by flow cytometry (^*^P<0.05; n=3)

To determine whether transcriptional inhibition of lncRNA-UCA1 affects its biological function, we measured cell viability using MTT assay. The results indicated that C/EBPα siRNA decreased the viability of the BLZ-211 cells (Fig. 6A). In order to validate whether the decreased cell viability via the reduction of C/EBPα is mediated through the reduction of lncRNA-UCA1, we used BLS-211 cells as control cells since they lack lncRNA-UCA1 expression. There was no obvious change in BLS-211 cell viability (Fig. 6B) following C/EBPα silencing. We also observed a significant promotion in apoptosis of the BLZ-211 cells induced by C/EBPα siRNA while no change was displayed in the BLS-211 cells (Fig. 6C and D). These results suggest that lncRNA-UCA1 is activated by C/EBPα, and thus, is involved in the regulation of bladder cancer cell growth and apoptosis.

## Discussion

Several recent reports have shown that lncRNAs play important roles in many physiological and pathological processes, particularly in carcinogenesis. lncRNAs have oncogenic or tumor-suppressive effects, and their dysregulation is found throughout the entire processes of generation, development and metastasis of cancers. Blocking the expression of oncogenic lncRNAs or activating the expression of tumor-suppressor lncRNAs could reverse the invasive and metastatic features of human cancer cells, indicating the potential versatility of lncRNAs as prognostic biomarkers and therapeutic targets for a diverse group of cancers (18,19). Among these lncRNAs, lncRNA-UCA1 particularly aroused our attention due to its significant upregulation in bladder cancer, which is closely associated with the proliferation, metastasis and drug resistance of bladder cancer cells. A recent study showed that lncRNA-UCA1 in urinary sediments is a highly specific and sensitive biomarker for the diagnosis and prognosis of bladder cancer (9). Consequently, the abnormal upregulation of lncRNA-UCA1 may contribute to the development and metastasis of human bladder carcinoma, while the underlying molecular mechanism for lncRNA-UCA1 dysregulation remains to be elucidated. Therefore, through bioinformatic analysis, we discovered that there are several potential transcription factor binding sites in the lncRNA-UCA1 core promoter, which indicate that these transcription factors may be involved in the regulation of lncRNA-UCA1 expression.

Highly upregulated in liver cancer (HULC) is an lncRNA uniquely overexpressed in liver cancer and plays a pivotal role in hepatocarcinogenesis (20). A cAMP responsive element binding protein (CREB) binding site is found in the HULC promoter (21). Bound and activated by p53 at the large intergenic non-coding RNA-p21 (lincRNA-p21) promoter region, lincRNA-p21 serves as a transcription repressor in the p53 pathway (22). These data are typical examples of the regulation of lncRNA activity by common transcription factors via binding with the promoters of lncRNAs. In the present study, we demonstrated that C/EBPα binds with the lncRNA-UCA1 core promoter (Fig. 2A and B). We also showed that C/EBPα modulates lncRNA-UCA1 expression (Figs. 4D and 5C). Our future research, extensive and more concrete, will focus on exploring other regulatory factors that influence lncRNA-UCA1 expression in bladder cancer, including DNA methylation, histone modification and specific microRNA expression.

Similar to other common transcription factors, C/EBPα is a ubiquitously expressed transcription factor that activates the transcription of various genes by interacting with the C/EBPα binding motif in respective promoters. A recent study reported a C1797G polymorphism in the protein coding gene of murine double minute 2 (MDM2) promoter of bladder cancer, and that the C to G substitution enhances the affinity of C/EBPα to this region in the MDM2 promoter (23). Intriguingly, we also found that the C/EBPα binding motif contains a base transversion (A231G) in T24 cells, and ChIP results showed that the brightness of the C/EBPα bands in T24 cells was significantly higher than that in the other two cell lines, which may be attributed to the base mutation in the C/EBPα binding motif (Fig. 2B and C) and this specific point mutation can affect the binding affinity of C/EBPα to the lncRNA-UCA1 promoter. Although our results indicated that the mutation (A231G) increased the transcriptional activity of lncRNA-UCA1, how it regulates lncRNA-UCA1 RNA levels remains unclear and warrants further research.

Our previous study showed that silencing of lncRNA-UCA1 expression in BLZ-211 cells resulted in the decrease in the expression of several cell cycle-associated genes, particularly encoded p300 and its co-activator CREB. Furthermore, lncRNA-UCA1 was found to regulate cell proliferation by activating CREB protein through the PI3-K/AKT signaling pathway (11). The PI3-K/AKT signaling pathway has been proven to play a critical role in cellular growth and apoptosis, and also serves as a therapeutic target in human cancers. In the present study, we confirmed that C/EBPα regulates lncRNA-UCA1 expression to increase cell viability and reduce cell apoptosis *in vitro*. Therefore, it is possible that the regulation of lncRNA-UCA1 expression by C/EBPα in manipulating cell viability and apoptosis may also be conducted via the PI3-K/AKT signaling pathway, and this hypothesis will be confirmed in forthcoming studies

In conclusion, our research reveals a novel mechanism for the upregulation of lncRNA-UCA1 expression in bladder cancer cells. These findings demonstrate that transcription factor C/EBPα is bound to the binding site in the core promoter of lncRNA-UCA1, leading to the activation of lncRNA-UCA1 transcription. The transcriptional activation of lncRNA-UCA1 by C/EBPα also contributes to the increased viability and decreased apoptosis of bladder cancer cells. Clinical studies aimed at assessing the association between lncRNA-UCA1 transcriptional regulators and pathological parameters of bladder carcinoma tissues are needed for confirming our results and for exploring the potential clinical application of lncRNA-UCA1 as a therapeutic target for bladder cancer.

## Figures and Tables

**Table I:** Primer, probe and siRNA list.

mRNA/gene promoter	Sequence (5′–3′)	Experimental use
UCA1	CTCTCCATTGGGTTCACCATTC	
	GCGGCAGGTCTTAAGAGATGAG	Real-time PCR
C/EBPα	ATTGGAGCGGTGAGTTTG	
	TTGGTGCGTCTAAGATGAG	Real-time PCR
β-actin	TCCCTGGAGAAGAGCTACGA	
	AGCACTGTGTTGGCGTACAG	Real-time PCR
UCA1 promoter (C/EBPα)	TCTCAGGCTGTCCTCTGGGAAG	
	TGTAGGCCACCTGGACATATATGTG	ChIP-PCR
UCA1 promoter (C/EBPα)	CATCACCCTGTAACAGGGAACTGTCAGG	
	CCTGACAGTTCCCTGTTACAGGGTGATG	Site-directed mutagenesis
UCA1 promoter (A231G)	CCCTGTAACGTTTCCAGAAGGGAACTGTCAGG	
	CCTGACAGTTCCCTTCTGGAAACGTTACAGGG	Site-directed mutagenesis
C/EBPα probe	CAGTTCCCTTTGGAAACGTTACAGGG	
	CCCTGTAACGTTTCCAAAAGGGAACTG	EMSA
C/EBPα mutant probe	CAGTTCCCTAACTTGAGCGTTACAGGG	
	CCCTGTAACGCTCAAGTTAGGGAACTG	EMSA
siRNA control	UUCUCCGAACGUGUCACGUTT	
	ACGUGACACGUUCGGAGAATT	RNA interference
C/EBPα	siRNA ACGAGACGUCCAUCGACAUTT	
	AUGUCGAUGGACGUCUCGUTT	RNA interference

**Table II:** Bioinformatic software programs predicting the transcription factors that bind with the lncRNA-UCA1 core promoter region.

Transcription factors	Bioinformatic software programs	Predicted results
C/EBPα (CCAAT/enhancer binding protein α)	AliBaba, MatrixCatch, Patch, TESS, TFSEARCH	+++++
CREB (cAMP responsive element binding protein)	AliBaba, Patch, TESS, TFSEARCH	++++
GATA-1 (globin transcription factor 1)	AliBaba, Patch, TESS, TFSEARCH	++++
c-MYB (myelocytomatosis viral oncogene homolog)	MatrixCatch, Patch, TESS, TFSEARCH	++++
SP1 (sequence-specific transcription factor 1)	AliBaba, Patch, TESS	+++

+, indicates the number of programs that predicted this transcription factor.

**Table III:** Bioinformatic software programs predicting the C/EBPα binding sites in the lncRNA-UCA1 core promoter region.

Bioinformatic software	Number of C/EBPα binding sites	Position (strand)	Matrix sequence
AliBaba	Three binding sites	−335 bp – 326 bp (+)	GCAGCTTGTG
		−282 bp – 273 bp (+)	GGAAAAGATA
		−264 bp – 255 bp (+)	GGCTGAGGTC
MatrixCatch	One binding site	−303 bp – 277 bp (+)	CAGTTCTGAAACCAGGACCAGGAAAA
Patch	Two binding sites	−302 bp – 294 bp (−)	TTCAGAAAT
		−238 bp – 233 bp (+)	TTTCCA
TESS	Two binding sites	−301 bp – 292 bp (+)	GTTCTGAAAC
		−238 bp – 229 bp (+)	TTTCCAAAA
TFSEARCH	One binding site	−241 bp – 228 bp (+)	ACGTTTCCAAAAG
